# Prolonged standing behaviour in people with joint hypermobility syndrome

**DOI:** 10.1186/s12891-021-04744-1

**Published:** 2021-12-01

**Authors:** Alexander Vernon Bates, Alison H. McGregor, Caroline M. Alexander

**Affiliations:** 1 Department of Surgery and Cancer, Imperial College London, London, UK; 2 Department of Therapies, Imperial College Healthcare NHS Trust, Charing Cross Hospital, London, W6 8RF UK

**Keywords:** Joint hypermobility syndrome, Hypermobile Ehlers Danlos syndrome, Prolonged standing, Joint laxity

## Abstract

**Background:**

Joint Hypermobility Syndrome (JHS) is a rare Heritable Disorder of Connective tissue characterised by generalised joint laxity and chronic widespread pain. Joint Hypermobility Syndrome has a large impact on patients’ day to day activities, and many complain of symptoms when standing for prolonged periods. This study investigates whether people with JHS exhibit the same behaviours to deal with the effects of prolonged standing as people with equal hypermobility and no pain, and people with normal flexibility and no pain.

**Methods:**

Twenty three people with JHS, 22 people with Generalised Joint Hypermobility (GJH), and 22 people with normal flexibility (NF) were asked to stand for a maximum of 15 min across two force-plates. Fidgets were counted and quantified using a cumulative sum algorithm and sway parameters of the quiet standing periods between fidgets were calculated.

**Results:**

Average standing time for participants with JHS was 7.35 min and none stood for the full 15 min. All participants with GJH and NF completed 15 min of standing. There were no differences in fidgeting behaviour between any groups. There was a difference in anteroposterior sway (*p* = .029) during the quiet standing periods.

**Conclusion:**

There is no evidence to suggest people with JHS exhibit different fidgeting behaviour. Increased anteroposterior-sway may suggest a muscle weakness and strengthening muscles around the ankle may reduce postural sway and potentially improve the ability to stand for prolonged periods.

## Background

JHS is a Heritable Disorder of Connective Tissue characterised by joint hypermobility and chronic pain, along with a suite of other articular and extra-articular symptoms [1]. It is a rare condition affecting approximately 1 in 5000 to 20,000 [2]. However, importantly the prevalence in healthcare is much higher; 39% in at a UK pain clinic [3], and 58% of females and 29% of males in a West-London general rheumatology clinic [4]. Interestingly in many people hypermobile joints are not associated with symptoms. Approximately one in five people have four or more hypermobile joints and often there are no (or at least very few) symptoms; the hypermobility may even be an asset in certain performing arts and sports [1]. The term Generalised Joint Hypermobility (GJH) can be used to define such patients. GJH is classified using the Beighton Score, where nine joints are tested for hypermobility and if four or more are hypermobile then the person scores positively as GJH [1]. JHS is classified using the Brighton Criteria [5]. The Brighton Criteria incorporates the Beighton Score with major and minor features of the syndrome. In 2017 the term JHS was superseded by Hypermobility Spectrum Disorder and Hypermobile Ehlers Danlos Syndrome [6, 7]. In this paper we use the terms JHS since this research was conducted prior to the new terms being proposed.

Prolonged standing is a common everyday activity which is normally performed secondary to another; for example, queueing, chatting, or working. Prolonged standing is demanding and can cause a wide range of both short and long-term effects. Physical symptoms include an increase in blood pressure causing venous distension (swelling of the blood vessels), superficial venous insufficiency, occlusion and pooling [8, 9], lower back pain [10], muscle fatigue [11], joint compression [12] and vertigo [13]. There are also psychological factors such as increased tension, mental fatigue, and stress [14, 15]. During prolonged standing people perform behaviours which are thought to counteract these effects. These behaviours include rapid changes of posture (i.e. “fidgets”), asymmetrical loading, and continuous low-amplitude body sway [13, 16]. These behaviours are thought to alleviate the symptoms of prolonged standing by several mechanisms. Carlsoo *et al.* [16] has proposed that varying the muscles and body structures used to support bodyweight allow muscles to relax and recover. Alexander [17] proposed that changes in position, or ‘fidgeting’, was a method to reduce joint pressure by circulating synovial fluid in the joints.

People with JHS complain specifically that prolonged standing is challenging, so much so that ‘standing for more than 30 minutes’ is included within the Bristol Impact of Hypermobility Questionnaire [18]. They have several features that could explain why they struggle; muscle weakness [19–22]; fatigue [20, 22–24]; impaired balance [25, 26]; impaired proprioception [26]; venous insufficiency and varicose veins [8]; pain, particularly in the knees and lower back [27, 28]; and hypermobility [29]. Further, an impact of chronic pain could lead to changes in neuromuscular control, which has been reported in hypermobile cohorts [30, 31], and might result in differences to the control of balance. We have only found one paper which explored the impact of prolonged standing in people with GJH; it concentrated on long-term vascular impact and found that hypermobility was a risk factor in developing venous insufficiency [8]. An investigation of prolonged standing behaviour could help build a picture of why this is such a problematic task; identifying differences between JHS and symptom-free behaviour could inform clinicians of potential areas to direct treatment. It is important to note that there is a gap in the current knowledge surrounding JHS as commonly studies compare a JHS group to a normal flexibility control group alone rather than also including a group with GJH [25]. This means that it is not clear whether differences between cohorts are due to hypermobility per se, or other features of JHS. To address this gap, in this paper we investigate prolonged standing behaviour of a group of JHS individuals compared to GJH and NF control groups. We hypothesise that prolonged standing behaviour will differ, namely that people with JHS will perform fewer behaviours that address the detrimental effects of prolonged standing.

## Methods

### Participants

Ethical approval was granted by NRES London-West Ethics Committee. We obtained informed and written consent from all participants. We defined hypermobility as a positive Beighton Score (4 or more hypermobile joints). The GJH group were classified as a positive Beighton Score and a negative classification of JHS using the Brighton Criteria. JHS were classified using the Brighton Criteria [1] (which also incorporates a positive Beighton Score). Inclusion criteria for the NF group was a Beighton Score < 4, and neither knee being hypermobile. Exclusion criteria for all groups were any history of lower limb surgery, and neurological or medical conditions not associated with JHS. GJH and NF participants were excluded if they had lower limb pain. This study was part of a wider investigation into movement in people with JHS which included balance reactions [32], gait, and stair climbing. The overall sample size for the wider investigation was based on kinematic outcome measures and informed by a previous study of hypermobile movement [33]. However, kinematic outcome measures are not a factor in this study and we could find no information on fidgeting behaviour in hypermobile cohorts on which to base a sample size calculation.

JHS participants were recruited from Ehlers-Danlos Support UK, The Hypermobile Syndromes Association, and patients from a London NHS Hospital. GJH and NF participants were recruited from posters displayed in the hospital and local area.

### Test procedure

We asked participants to stand over two Kistler force plates (Kistler Instruments Corp., Amherst, USA) sampling at 50 Hz. A Vicon system (Oxford Metrics Ltd., Oxford, UK) was used to record force plate data. We instructed participants to stand with one foot on each of the force plates and told them that they could change position as they wished, but they must not place both feet on a single force plate. Participants rated their joint pain on a numeric scale from 0 to 10 at the start of the standing. We chose a numeric rating scale as its reliability, validity, and sensitivity are well established [34]. Participants were asked to stand on the force plates for a maximum of 15 min, or until their pain score had increased by two points, which is generally considered to be a clinically meaningful change [35, 36] . This stop criteria was established in order that the participants’ pain was not aggravated more than necessary to discover any differences between the groups. During testing no JHS participants stood for more than 15 min, therefore we set a maximum standing time for GJH and NF groups of 15 min. Participants were instructed to tell the researcher if they felt that their pain had increased by two points. Since prolonged standing is usually performed secondary to another task, we asked participants to watch a documentary on a tablet computer placed 50 cm in front of them. This also served to keep orientation constant between participants. The same documentary was shown to all participants.

### Data analysis

When a participant changes position, they move one of their feet and shift their bodyweight, this generates a change in the vertical force signal from the relevant force plate. Detecting changes in position can therefore be thought of as detecting periods of change in the force plate time series. We used a cumulative sum algorithm which has been used and described in other studies of prolonged standing [37]. As a brief overview the CUSUM algorithm relies on two user inputs; the magnitude of the change to be detected, and a ‘drift’ variable to prevent false positives caused by small-scale changes. The algorithm calculates the cumulative sum of both the positive and negative changes in a time series and compares the change to the threshold, when the threshold is passed a change is detected and the cumulative sum resets to zero (see Prado *et al.* [37] for a detailed description).

Fidgets were quantified at three bodyweight thresholds; greater than 50%, 25–50%, and 10–25% and were normalised to fidgets per minute. These thresholds were determined by visual inspection of a selection of participants’ data. The greater than 50% threshold was included as it contains the largest fidgets; in theory, the largest fidget to be recorded would be 100% bodyweight, a shift from standing with all weight on one leg to all weight on the other leg. The 10% cut off was selected as it was the smallest fidget able to be visually separated from the vertical component of the force signal (Fz) generated by sway during quiet standing periods. The remaining fidget of 25–50% was selected to capture ‘medium’ size fidgets. After fine tuning the algorithm to correlate with visual interpretation, the drift variable was set as 0.01% of bodyweight. Note that these thresholds are the same as Prado *et al*. [37], thus providing some assurance.

Of interest were other variables commonly used in posturography. These were anterioposterior (AP) sway, the standard deviation of COP in the anterioposterior axis; mediolateral (ML) sway, the standard deviation of the COP in the mediolateral axis; sway area, the elliptical area containing 95% of the COP points; and sway velocity; the velocity at which the COP moves in the AP/ML plane. These variables are traditionally extracted when a participant is asked to stand still, for example during the Romburg test, and would be affected by the changes of posture during prolonged standing. Therefore, periods of ‘quiet standing’ were extracted, i.e. the periods between fidgets where participants had adopted a relatively static posture. To achieve this, the positions of fidgets of 10% bodyweight or more were identified using the CUSUM formula, data ±1.5 s from the fidget was removed for the analysis of quiet standing. This left a time series for each variable containing only periods of quiet standing.

Force plate data was exported from Vicon and processed in MatLab (The MathWorks, Natick, United States). Statistical analysis was performed in SPSS version 24. Data was tested for normality using a Shapiro-Wilk test. A one-way ANOVA was used to investigate the effect of group on variables that were normally distributed. A Kruskal-Wallis test was used to compare outcome measures that were not normally distributed. Effect size for Kruskal-Wallis tests were computed as the eta squared based on the H-statistic [38]. Where significant differences were found, pair-wise comparisons were made using Mann-Whitney U tests. Corrections for multiple comparisons were performed using the Bonferroni method. Significance was set at *p* < .05.

## Results

The demographics of the participants reveal no significant differences across age, height or BMI (Table 1). No participant reported an adverse event during or following the testing session.

**Table 1:** Mean ± standard deviation

	JHS (n = 23)	GJH (n = 23)	NF (n = 22)
Age (years)	33 ± 9	28 ± 6	28 ± 5
Sex (f/m)	20/3	19/4	16/6
Height (cm)	169 ± 8	169 ± 10	172 ± 8
BMI	25.5 ± 5.6	22.9 ± 4.4	22.0 ± 2.8
Beighton Score^a^	6.8 ± 2.1	6.6 ± 1.3	0.3 ± 0.7
Visual analogue score knee pain, scored from 0–10^a,b^	3.2 ± 2.2	0.0 ± 0.0	0.0 ± 0.0

^a^indicates a significant difference between JHS and NF groups,

^b^indicates a significant difference between JHS and GJH groups. Significance was set at *p* < .05

Based on 22 participants per group, post-hoc power calculations gave a power of 0.8 with an effect size of 0.4, and a power of 0.95 with an effect size of 0.5.

All outcome measures were not normally distributed. Table 2 shows the median and interquartile range for each outcome measure. The standing time represents the time taken for a participant’s pain score to increase by two points on a pain numerical rating scale, which marks the end of the task (the pain score of participants who stood for 15 min did not reach two points above their starting score). There was a significant difference in standing time between groups (*p* < 0.001, effect size = 0.664). For pair-wise comparisons, JHS stood for a significantly shorter time than NF (*p* < .001) and GJH (*p* < .001), and there was no significant difference between GJH and NF (*p* = 1.000). There was also a significant difference between groups in starting pain (*p* < .001); JHS had significantly greater starting pain than both GJH and NF groups (*p* < .001 in both cases).

**Table 2:** Median (interquartile range) of outcome measures for each group

	JHS (n = 23)	GJH (n = 22)	NF (n = 22)
Standing time (minutes)	7.35 (4.15–14.18); *P* < 0.001 vs GJH; *p* < 0.001 vs NF	15; *p* < 0.001 vs JHS	15; *p* < 0.001 vs JHS
Starting pain (0–10)	1 (0–3)	0	0
Fidgets (>50% bodyweight)	1.19 (0.24–2.06)	0.41 (0.27–1.41)	0.26 (0–1.28)
Fidgets (25–50% bodyweight)	2.12 (0.79–4.68)	1.04 (0.73–3.1)	0.7 (0.22–1.79)
Fidgets (10–25% bodyweight)	5.8 (1.4–11.21)	3.87 (2.75–7.59)	2.39 (1.34–6.41)
AP sway (mm)	30.45 (17.82–39.54); *p* = .041 vs NF	23.79 (17.38–27.1)	16.32 (10.34–28.75); *p* = .041 vs JHS
ML sway (mm)	32.51 (16.76–56.96)	30.29 (17.52–50.12)	16.87 (9.58–66.5)
Sway area (cm^2^)	170.1 (48.63–419.02)	109.94 (57.26–162.73)	48.31 (18.91–249.26)
Sway velocity (mm/s)	65.11 (25.92–98.74)	94.35 (89.14–118.3)	88.09 (73.79–117.27)

Interquartile ranges of Standing time and Starting are not listed for GJH and NF groups as they all completed 15 min of standing and did not have any joint pain. Significance was set at *p* < .05

**Fig. 1:**
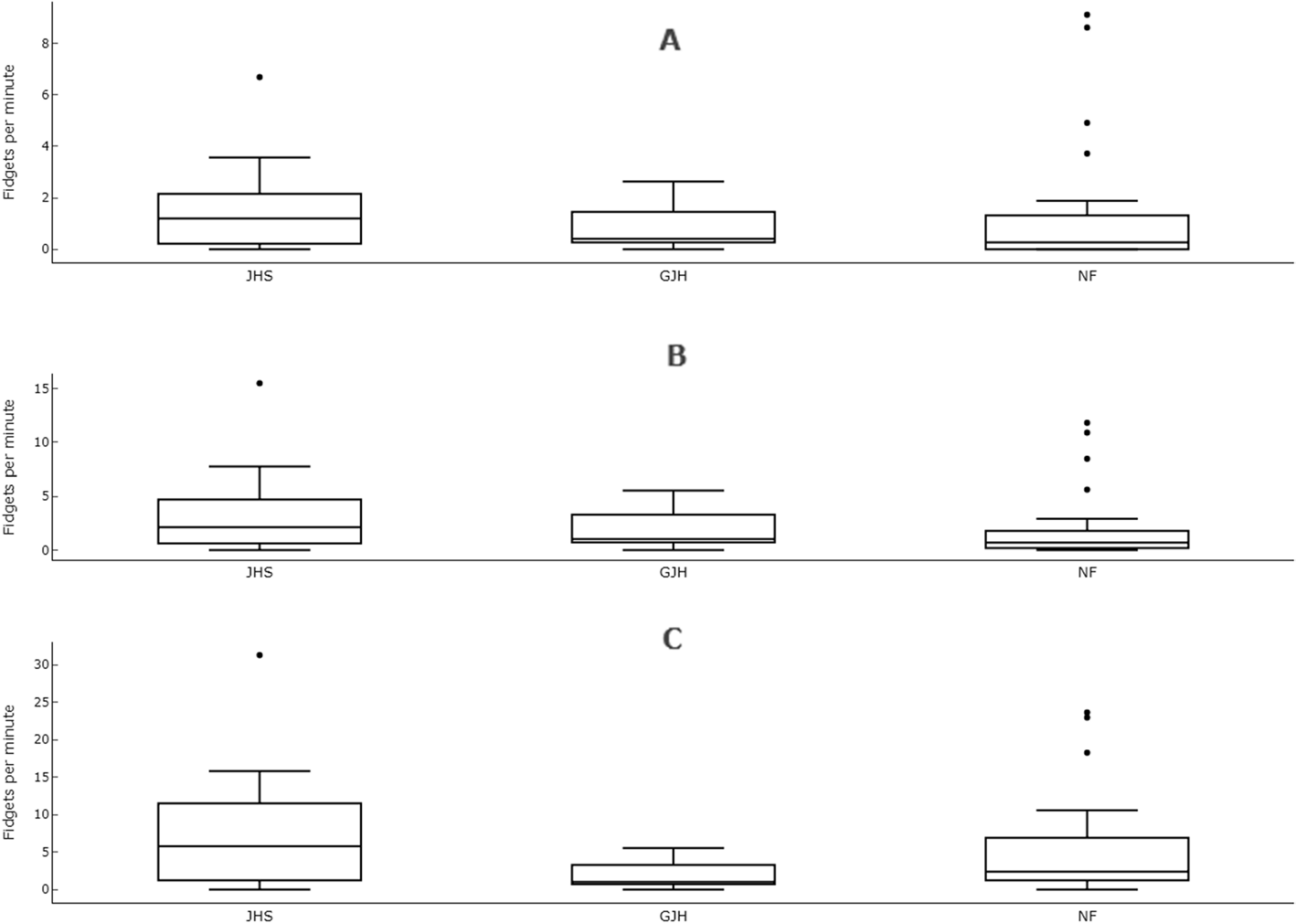
Fidgets per minute for each group at the different fidget magnitudes. **A** Fidgets at over 50% bodyweight, **B** Fidgets 25–50% bodyweight, **C** Fidgets 10–25% bodyweight. Horizontal lines indicate median values; boxes show the 25th to 75th percentile range; vertical lines show adjacent values within 1.5 interquartile range of the 25th and 75th percentile; points denotes outliers

Although generally the JHS group showed a greater median number of fidgets and a wider variability in the number of fidgets at each bodyweight magnitude, these differences were not significant (Fig. 1). In the NF group there were several outliers to the distribution. All data, including outliers, were included in analysis.

**Fig. 2:**
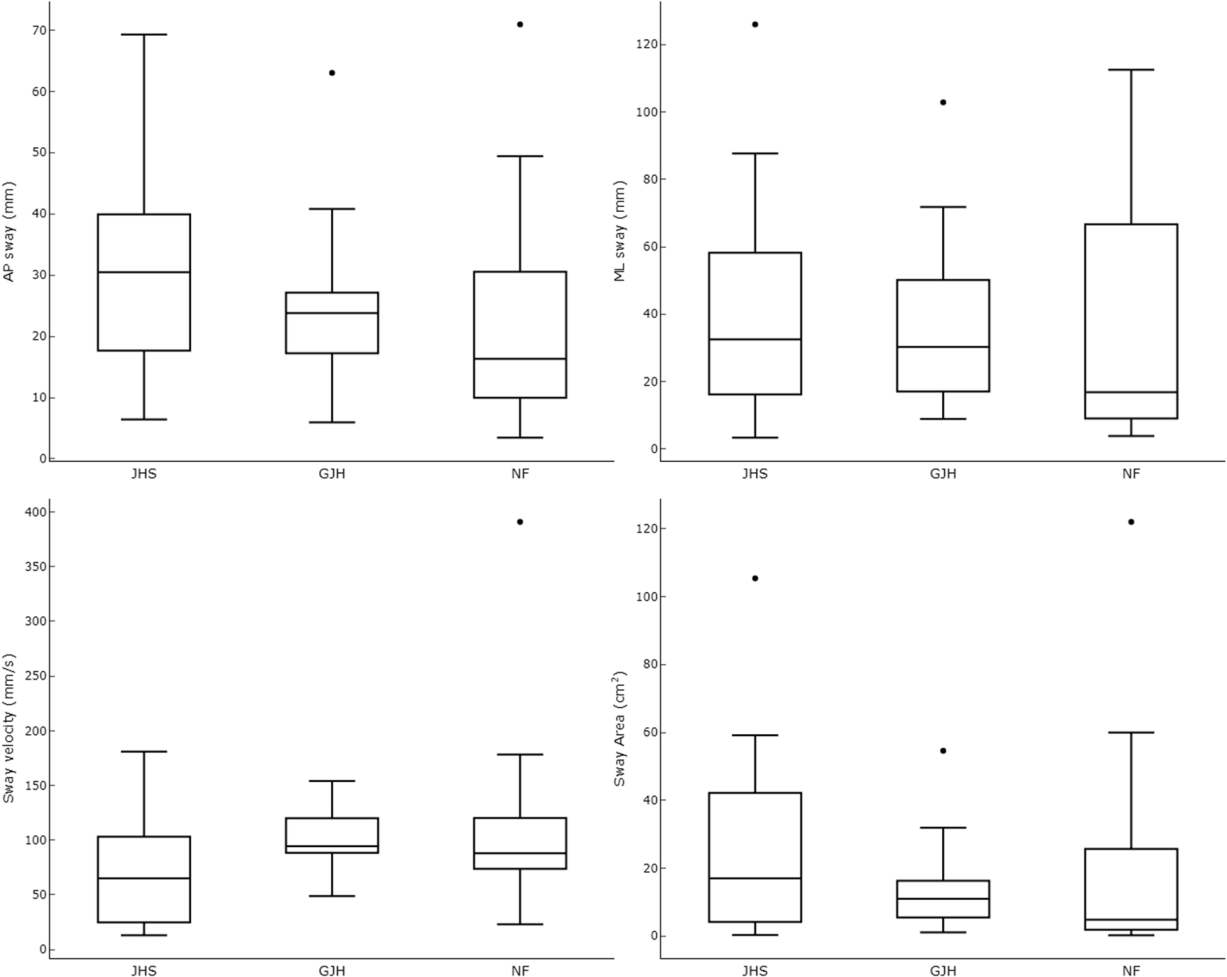
Boxplot showing sway parameters during prolonged standing. Anterioposterior (AP), Mediolateral (ML) sway, Sway velocity, and Sway area for each cohort. Parameters calculated during quiet standing. Horizontal lines indicate median values; boxes show the 25th to 75th percentile range; vertical lines show adjacent values within 1.5 interquartile range of the 25th and 75th percentile; markers denote outliers

During the quiet standing periods there was no significant difference between any of the groups in ML sway, sway area, or sway velocity (Fig. 2). There was a significant difference between groups in AP sway (*p* = .029, effect size = 0.0571). For pair-wise comparisons, there was a significant difference between JHS and NF groups (*p* = .041), and no significant difference between JHS and GJH (*p* = .190) or NF and GJH (*p* = 1.00).

## Discussion

This is the first study of prolonged standing behaviour in hypermobile individuals and the aim was to see if there were differences in standing behaviour, which might give some insight as to why people with JHS find prolonged standing so discomforting. The results confirm that people with JHS struggle to stand for as long as people who are equally hypermobile without pain (GJH group) or have normal flexibility without pain (NF group). That is, the median standing time of the JHS group was 7.35 min, with a good effect size of 0.7 and was significantly less than both GJH and NF groups. Since participants were asked to stop once their pain had increased by two points on a numerical scale, this shows that the JHS group found the task aggravating. The results of this study emphasise how difficult people with JHS find prolonged standing.

We measured fidgets and sway. A reduction in the number of fidgets has been linked to fear of falling, lower stability, and a lack of somatosensory information [10, 14, 37]. It is interesting that although the wide spectrum of features and symptoms that could influence standing behaviour; JHS individuals fall frequently [26], have an increased fear of falling [26], impaired balance [26, 39], and reduced proprioception in the lower limbs [40–42]; that these impairments do not manifest as a change in fidgeting behaviour during prolonged standing. Although the differences were not significant, the JHS median fidgets were greater than other groups at each of the fidget magnitudes measured. Although the median and interquartile range of NF fidgets was lower than JHS, there were several outliers in the NF group who performed a relatively large number of fidgets; outside the range of the JHS and GJH groups. We decided to include all data, including outliers, in the statistical analysis as it is possible that these outliers reflect the underlying variability of the population (removing them would be arbitrary as there is insufficient data on fidgets in people with NF). However, if these are genuine outliers, removing them would enhance the power of the study and may better show differences between groups. Considering the functional purpose of fidgets described previously, it may be that this data provides a hint that JHS individuals perform more fidgets to relieve their symptoms of pain and fatigue, or to gather more visual information about the environment.

It is important to note that an impact of people with JHS standing for a shorter time than control groups is that any change to fidgets or sway that could happen over time, is not captured. Freitas *et al*. [13] found that postural behaviours, including fidgets, changed significantly with standing duration; particularly relevant is that standing behaviour during the first 10 min of standing differs to the last 10 min (i.e. within the time-frame of this study). Given that most JHS participants did not reach 10 min of standing time, it could be that differences between groups would be more observable in longer standing periods. Crucially, we stopped the activity when participant’s self-reported pain score increased by two points. This intervention meant that we were capturing fidgeting behaviour when pain raises to greater levels. Given fidgeting and increased sway can be a response to discomfort, it may therefore be that the outcome measures considered here would increase even more if they could have stood for longer.

JHS participants had significantly more anterioposterior sway compared to NF and GJH control groups, however the small effect size (0.06) may mean that this change might not be sufficiently clinically meaningful. Increased sway has been observed in JHS groups during periods of quiet standing and is thought to be indicative of impaired balance and reduced proprioception [26, 39]. The increased sway observed here could be the observed impaired balance manifesting during prolonged standing. Alternatively, increased sway has been cited as a way of people alleviating the fatigue of prolonged standing [13], the increase here may be JHS individuals increasing sway to deal with becoming fatigued more quickly, perhaps due to muscle weakness of the anterior and posterior compartment musculature in the lower leg. Another reason for fatigue could be that it is centrally mediated. People with JHS have been reported to suffer with central fatigue during tasks that do not fatigue people with normal flexibility [43]. It should also be remembered that there were no differences in fidgets between cohorts. If the increase in sway was a mechanism of dealing with fatigue, then it would be expected fidgets also would increase, which lends weight to the sway observed here being related to balance or muscle weakness. It suggests that clinicians might advise concentrating on improving strength around the ankle and increasing the number of fidgets to alleviate symptoms.

## Limitations

A limitation is that the project may be inappropriately powered as the initial sample size was generated for a different primary outcome as part of a wider investigation into kinematics and kinetics in people with JHS. In the present study, we did not calculate a sample size due to lack of information about fidgeting behaviour in JHS and what a meaningful effect size would be. With that in mind, effect-sizes are calculated where differences between groups are significant. However, we could find no information about what level effect = −size is clinically meaningful in the parameters measured. Furthermore, due to the lack of a sample size calculation, there is a risk of Type II error. Another potential limitation of this study was that participants were instructed to watch, and were therefore orientated by, the tablet computer they were watching. This means that AP and ML sway is not the exact same definition as other studies investigating quiet standing (where the position of the feet and orientation of participant are more strictly defined).

## Conclusion

Although this study found no evidence of people with JHS exhibiting different fidgeting behaviour during prolonged standing, people with JHS did show increased anteroposterior-sway. This may suggest a muscle weakness; strengthening muscles around the ankle may reduce postural sway and potentially improve the ability to stand for prolonged periods.

## Data Availability

The datasets used and/or analysed during the current study are available from the corresponding author on reasonable request.

## Abbreviations

AP: Anteroposterior
Fz: Vertical component of the ground reaction force
GJH: Generalised Joint Hypermobility
JHS: Joint Hypermobility Syndrome
ML: Mediolateral
NF: Normal Flexibility
